# The rCC16 Protein Protects Against LPS-Induced Cell Apoptosis and Inflammatory Responses in Human Lung Pneumocytes

**DOI:** 10.3389/fphar.2020.01060

**Published:** 2020-07-14

**Authors:** Jinle Lin, Jiemei Li, Min Shu, Weigang Wu, Wenwu Zhang, Qingli Dou, Jian Wu, Xiaobin Zeng

**Affiliations:** ^1^ Department of Emergency Medicine, Shenzhen Baoan First People’s Hospital, Nanfang Medical University, Shenzhen, China; ^2^ Department of Respiratory and Critical Care Medicine, Guangdong Provincial People’s Hospital, Guangdong Academy of Medical Science, Guangzhou, China; ^3^ Center Laboratory of Longhua Branch and Department of Infectious Disease, Shenzhen People’s Hospital (The Second Clinical Medical College, Jinan University; The First Affiliated Hospital, Southern University of Science and Technology), Shenzhen, China; ^4^ Emergency Department, Huazhong University of Science and Technology Union Shenzhen Hospital, Shenzhen, China; ^5^ Guangdong Provincial Key Laboratory of Regional Immunity and Diseases, Medicine School of Shenzhen University, Shenzhen, China

**Keywords:** CC16, Acute Respiratory Distress Syndrome, LPS, inflammation, apoptosis

## Abstract

**Objective:**

Our previous clinical study showed that low lung levels of CC16 strongly influence the occurrence and development of ARDS. The aim of the present study was to evaluate the therapeutic effect of rCC16 on LPS-induced inflammation in A549 cells and to determine its mechanism.

**Methods:**

Cell apoptosis and inflammation was induced by LPS stimulation. The cytotoxic effect of rCC16 was evaluated using the MTT assay. Cytokine levels were determined using enzyme-linked immunosorbent assays. The molecular mechanism of rCC16 was investigated by analyzing relevant signaling pathways.

**Results:**

The LPS treatment of A549 cells significantly decreased cell viability, increased the levels of the apoptotic proteins Bax, Bak and Cleaved Caspase-3, the secretion of inflammatory cytokines, and the expression levels of TLR4, p-NF/κB, MAPK proteins. While the levels of Bcl-2, p-AKT, p-mTOR, p-ERK1/2, NF/κB, p-AMPK, and p-p38 were significantly decreased in LPS-treated A549 cells. Our experimental results also confirmed that rCC16 inhibited LPS-induced apoptosis, promoted A549 cell proliferation by activating the PI3K/AKT/mTOR/ERK1/2 pathway, and inhibited the release of certain inflammatory factors, especially HMGB1, through dephosphorylation and inactivation of the TLR4/NF-κB/AMPK signaling pathways.

**Conclusion:**

These results highlight the potential utility of CC16 as an important cytokine for the prevention or treatment of inflammation and show that CC16 may play an important role in the future clinical treatment of ARDS.

## Introduction

Acute Respiratory Distress Syndrome (ARDS) is a life-threatening disease occurring in critically ill patients and has an estimated mortality of 40%–45%. ARDS is characterized by severe hypoxic respiratory failure, pulmonary infiltrates (Eworuke et al., 2018), and an overwhelming inflammatory response in the lung (Hudson et al., 2012). Although supportive therapies for ARDS have been rapidly developed, the mortality rate of ARDS has not greatly improved in recent years (Ware and Matthay, 2000). Therefore, exploration of the molecular mechanisms of ARDS and discovery of novel therapeutic options have become research hotspots.

One ARDS research focus on the Clara secretory cell protein (CC16), an anti-inflammatory factor native to the lung. CC16 is a 16-kDa homodimeric protein predominantly secreted by nonciliated airway epithelial cells (Nord et al., 2000). CC16 can suppress the release of pro-inflammatory cytokines, such as TNF-α, IL-1β, and IFN-γ, in the lungs (Dierynck et al., 1996); therefore, reduced CC16 levels in the bronchial epithelium or sputum supernatants and reduction in the numbers of CC16-positive epithelial cells in the small airways of asthmatics aggravate chronic lung inflammation (Pilette et al., 2001). Mice lacking CC16 show increased susceptibility and exaggerated inflammatory responses to hyperoxic or infectious agents (Snyder et al., 2010).

Our previous clinical research showed that CC16 can serve as a highly specific biomarker for the diagnosis and treatment of ARDS (Lin et al., 2018). In addition, increasing the CC16 content in the serum or lung tissues can inhibit the expression of various inflammatory factors, such as HMGB1, thereby reducing the inflammatory response (Pang et al., 2015). These findings indicate that CC16 is of great importance in maintaining lung homeostasis. In the present study, we investigated the lung-protective activity of rCC16 against LPS-induced cell apoptosis and inflammation, and we identified potential mechanisms.

## Materials and Methods

### Reagents

Recombinant CC16 protein was purchased from Proteintech (ag0758, Chicago, MA, USA), and LPS was obtained from Solarbio (813Q039, Beijing, China). Dulbecco’s modified Eagle’s medium (DMEM), fetal bovine serum (FBS), 100 U/ml penicillin, and 100 mg/ml streptomycin were purchased from HyClone (Logan, UT, USA). 4,6-Diamidino-2-phenylindole (DAPI, D9542) was purchased from Sigma-Aldrich (St. Louis, MO, USA). 3-(4,5-Dimethylthiazol-2-yl)-2,5-diphenyl tetrazolium bromide (MTT, M8180) was purchased from Solarbio (Beijing, China). Antibodies against mammalian target of rapamycin (mTOR, 2972), phosphorylated-mTOR (p-mTOR, 2974), serine/threonine kinase 1 (AKT, 4691), phosphorylated-AKT (p-AKT, 4060), extracellular regulated protein kinases (ERK1/2, 4695), phosphorylated-ERK1/2 (p-ERK1/2, 4370), caspase-3 (9664), Bcl-2 (15071), Bak (12015), Bax (2774), Cox 2 (12282), high mobility group box 1 protein (HMGB1, 6893), Toll-like receptor 4 (TLR4, 14358), phosphoinositide 3-kinase (PI3K, 4249), nuclear factor-κB (NF-κB, 8242), phosphorylated nuclear factor-κB (p-NF-κB, 3303), p38 mitogen-activated protein kinase (p38 MAPK, 8690), phosphorylated-p38 MAPK (p-p38 MAPK, 4511), adenosine monophosphate-activated protein kinase (AMPK, 5832), and phosphorylated-AMPK (p-AMPK, 50081) were obtained from Cell Signaling Technology (Danvers, MA, USA). Horseradish peroxidase (HRP)-conjugated rabbit anti-mouse IgG secondary antibody (ab6728) and anti-GAPDH antibody (ab8245) were purchased from Abcam (Cambridge, MA, USA). Alexa Fluor 488-conjugated goat anti-rabbit antibody was purchased from Invitrogen (Carlsbad, CA, USA).

### Cell Culture

Human A549 cells were purchased from the American Type Culture Collection (Manassas, USA). The cells were cultured in DMEM supplemented with 10% FBS, 100 U/ml penicillin, and 100 mg/ml streptomycin. All cells were cultured in an incubator at 37°C in 5% CO_2_. Adherent cells were digested every 2 days with 0.25% trypsin-EDTA (Life Technologies, Pitampura, India).

### Cell Viability Assay

MTT assays were used to measure cell viability. Briefly, 200 μl A549 cells at an initial density of 1.0 × 10^4^ cells/well were seeded in 96-well microplates and allowed to adhere for 24 h. The cells were then treated with LPS at different concentrations (0, 20, 40, 80, 100, and 150 μg/ml) for 3–48 h. After the exposure period, 100 μl 5 mg/ml MTT in DMEM was added to each well for 4 h. The MTT-containing medium was then removed, and 150 μl dimethyl sulfoxide was added to dissolve the formed formazan crystals. The absorbance was measured with spectrophotometric microplate reader at 590 nm.

### Determination of the Level of Cytokines

The levels of the cytokines Interleukin- 6 (IL-6, Item No. 13515), Interleukin- 8 (IL-8, Item No. 13147), Interleukin- 1β (IL-1β, Item No. 13089), Pulmonary Surfactant Associated Protein C (SP-C, Item No. 10571), Tumor Necrosis Factor-α (TNF-α, Item No. 11776), High Mobility Group Protein (HMGB1, Item No. 15639), Cyclooxygenase-2 (Cox-2, Item No. 10711), and Inducible Nitric Oxide Synthase (iNOS, Item No. 11708) were determined by enzyme-linked immunosorbent assays (ELISAs) following the manufacturers’ protocols (LunchangshuoBiotech, Xiamen City, Fujian Province, China). Briefly, the cells were collected after treatment for 3–12 h, washed twice with cold PBS, and resuspended at a concentration of 1 × 10^6^ cells/ml in 1× Binding Buffer. The detection range for IL-1β, IL-6, IL-8, and SP-C ELISA kits (JL, Jianglai Biological, Shanghai, China) was 1.0–200, 1.0–160, 1.0–120, and 1.0–40 pg/ml, respectively, and the limit of detection for all the kits was 1.0 pg/ml. The detection range for the TNF-α ELISA kit (JL, Jianglai Biological, Shanghai, China) was 10–640 pg/ml, and the limit of detection was 5.0 pg/ml. The detection range for the HMGB1 and Cox 2 ELISA kits (JL, Jianglai Biological, Shanghai, China) was 0.1–8 and 1.0–120 ng/ml, respectively, and the limit of detection for both was 0.1 ng/ml. The detection range for the iNOS ELISA kit (JL, Jianglai Biological, Shanghai, China) was 0.1–40 U/L, and the limit of detection was 0.1 U/L.

### Western Blot Analysis

Briefly, cell pellets were lysed in RIPA buffer on ice for 30 min. The lysates were centrifuged at 10,000 × g for 30 min at 4°C. Cytoplasmic and nuclear proteins were prepared according to the manufacturer’s instructions with a nucleoprotein and cytoplasmic protein extraction kit (P0028, Beyotime, Shanghai, China). Samples were then centrifuged at 15,000 × g for 10 min at 4°C. Protein concentrations were determined using a modified bicinchoninic acid (BCA) protein assay kit (P0010S, Beyotime, Shanghai, China). The protein samples were subjected to western blotting according to standard protocols. Briefly, proteins of different molecular weights were separated by SDS-PAGE and electrophoretically transferred to PVDF membranes. The membranes were blocked with 5% nonfat milk for 1 h at room temperature and then probed with the specific primary antibodies overnight at 4°C. The membranes were washed with PBS containing 0.1% Tween-20 and then probed with peroxidase-conjugated secondary antibodies for 1 h at 24°C. The protein bands were detected with by chemiluminescence (DNR Bio-Imaging Systems, Jerusalem, Israel). Signal intensity was quantified by densitometry with a Gel-pro Analyzer (Media Cybernetics, MD, USA). GAPDH was used as the protein loading control, and all experiments were performed in triplicate.

### Immunofluorescence Staining

Immunofluorescence staining was assessed to detect the subcellular distributions of Bax, Bcl-2, p-NF-κB/p65, and p-p38 MAPK, as previously described by Wan et al. (2019). Briefly, A549 cells pretreated with or without 0.2 mg/ml rCC16 for 3 h were grown on coverslips and then stimulated with 200 μg/ml LPS for 24 h. The cells were rinsed with PBS, fixed with 4% paraformaldehyde at 24°C for 20 min, incubated with PBS containing 0.25% Triton X-100 for membrane permeabilization, and then blocked with 5% BSA for 1 h. The cells were then incubated with specific primary antibodies overnight at 4°C, washed with PBS, and incubated with an Alexa Fluor 488-conjugated goat anti-rabbit antibody (1: 200) in the dark for 1 h. The cells were then stained with DAPI for 10 min and images were captured with a fluorescence microscope (CKX41-F32FL, Olympus, Japan).

### Statistical Analysis

Data were analyzed using SPSS version 17.0 software. The measured data are presented as the mean ± SD taken from three or more independent experiments. Comparisons between the control group, model group, and experimental group were performed by one-way ANOVA. Comparisons between groups were performed with a 2-tailed unpaired t-test. P values less than 0.05 were considered statistically significant.

## Results

### Effects of LPS on the Viability of A549 Human Lung Pneumocytes

**Figure 1:**
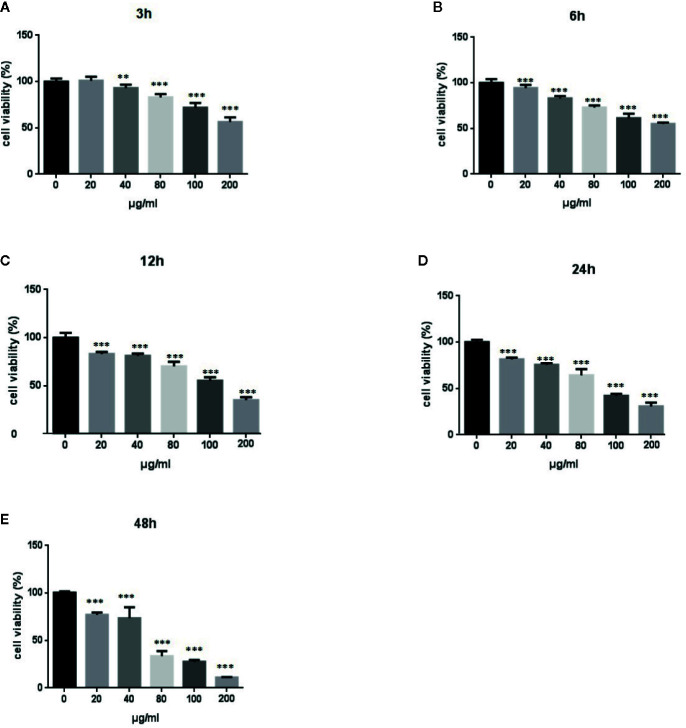
Effects of LPS at concentrations of 0–150 μg/ml on A549 cell viability. MTT assays show time- and-dose dependent A549 cell proliferation response to LPS treatment **(A–E)**. *p < 0.05, **p < 0.01, and ***p < 0.001 compared with the control group. Graphs show the mean ± SD of triplicate wells and represent three independent experiments.

We performed MTT assays of A549 cells to investigate the effect of LPS treatment on the viability of human lung pneumocytes (Standiford et al., 1990). As shown in **Figures 1A–E**, LPS treatment suppressed A549 cell viability in a dose- and time-dependent manner.

### LPS Induced A549 Cell Apoptosis Through the PI3K/AKT/mTOR/ERK1/2 Pathway

**Figure 2:**
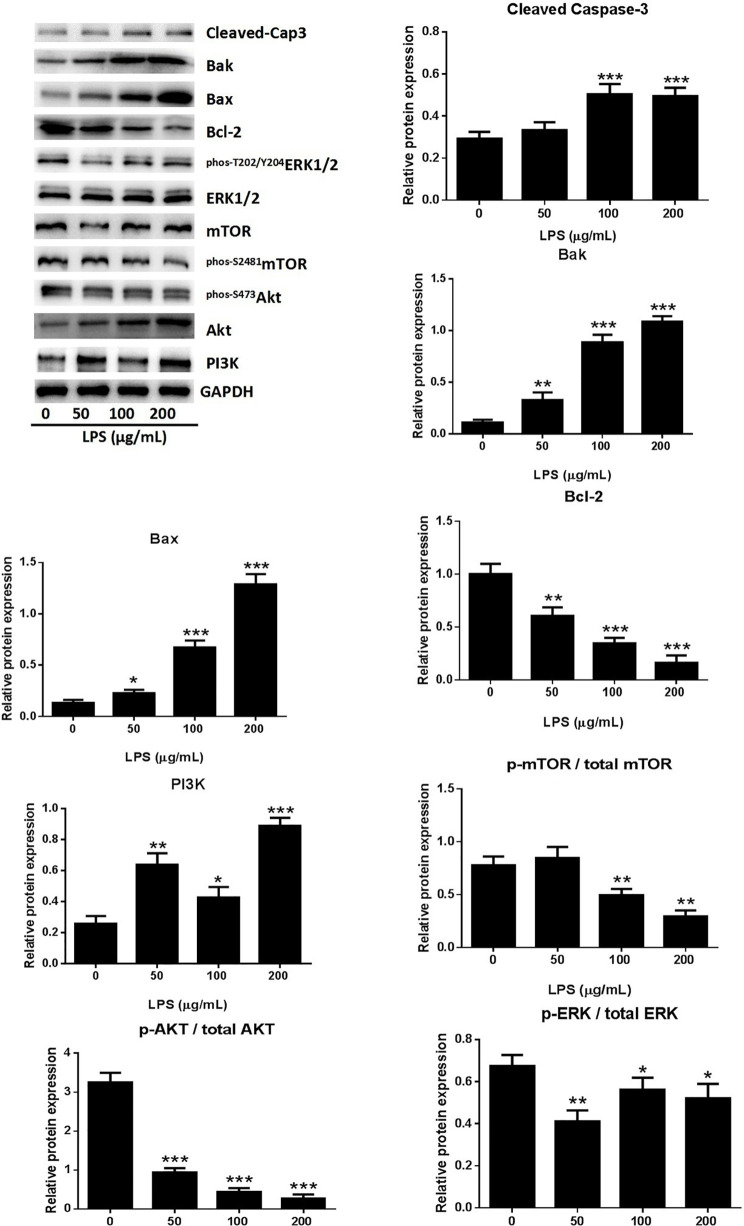
LPS induced A549 cell apoptosis through the PI3K/AKT/mTOR/ERK1/2 pathway. Western blot analysis and quantification of apoptosis-related proteins and PI3K/AKT/mTOR/ERK1/2 signaling-related proteins in A549 cells stimulated with various concentrations of LPS for 24 h. *p < 0.05, **p < 0.01, and ***p < 0.001 compared with the control group. Graphs show the mean ± SD of triplicate wells and represent three independent experiments.

A549 cell death occurred after stimulation with 0–200 μg/ml LPS. The form of cell death was determined by western blot analysis of the proteins of A549 cells treated with LPS. The expression of activated caspase-3 showed dose-dependent increases in response to LPS treatment (**Figure 2**), indicating that the LPS can induced cell apoptosis in human lung pneumocytes. The identity of the LPS-induced apoptosis mechanism was explored by assessing the protein levels of the anti-apoptotic protein B-cell lymphoma-2 (Bcl-2) and the pro-apoptotic proteins bcl-2 associated X protein (Bax) and bcl-2 antagonist killer (Bak) (Ge et al., 2019). Western blotting showed that LPS induced Bax and Bak expression and reduced Bcl-2 expression (**Figure 2**), suggesting that LPS triggers apoptosis in human lung pneumocyte cells by the intrinsic pathway.

The PI3K/AKT/mTOR pathway is critical for cell proliferation, growth, and survival and is constitutively activated in many types of cells (Yap et al., 2008). Examination of the activation of AKT and mTOR revealed significantly reduced levels of p-AKT, p-mTOR, and p-ERK1/2 in A549 cells after treatment with LPS (**Figure 2**), suggesting that LPS deactivated mTOR through an AKT-dependent pathway. The ERK protein is a significant upstream regulator of mTOR, and our assessment of ERK1/2 phosphorylation in A549 cells after LPS treatment revealed a dose-dependent downregulation of ERK1/2 activation by LPS (**Figure 2**). These results indicated that LPS induces A549 cell apoptosis and inhibits proliferation through PI3K/AKT/mTOR/ERK1/2 pathways.

### LPS Induced the Release of Inflammatory Cytokines in A549 Cells

**Figure 3:**
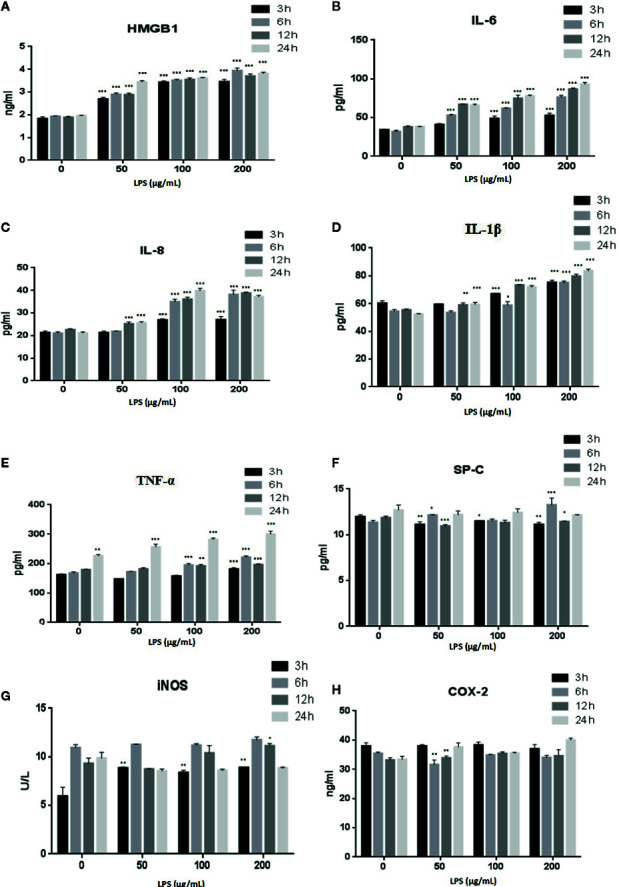
LPS induced release of the inflammatory cytokines HMGB1 **(A)**, IL-6 **(B)**, IL-8 **(C)**, IL-1β **(D)**, TNF-α **(E),** SP-C **(F)**, iNOS **(G)**, and COX-2 **(H)** from A549 cells. *p < 0.05, **p < 0.01, and ***p < 0.001 compared with the control group. Graphs show the mean ± SD of triplicate wells and represent three independent experiments.

LPS is a major component of the outer membrane of gram-negative bacteria and is often used to study cell inflammation (He et al., 2020). The effects of LPS treatment of A549 cells on the expression of inflammatory factors HMGB1, TNFα, IL-6, IL-8, IL-1β, SP-C, iNOS, and COX-2 were evaluated with ELISAs (**Figure 3**). The secretion of HMGB1 (**Figure 3A**) and IL-6 (**Figure 3B**) were increased in a time- and dose-dependent manner (p < 0.001). IL-8 (**Figure 3C**) (p < 0.001) and IL-1β (**Figure 3D**) (p < 0.001, or p < 0.01) also showed time- and dose-dependent releases following treatment with high doses of LPS or after prolonged treatment times. The levels of TNF-α (**Figure 3E**) decreased after a 24 h LPS treatment (50 μg/ml) (p < 0.001). Examination of other cytokines, such as SP-C (**Figure 3F**), iNOS (**Figure 3G**), and COX-2 (**Figure 3H**), revealed no consistent changes.

### LPS Induced Inflammatory Responses Through the MAPK/NF-κB/TLR4 Pathway

**Figure 4:**
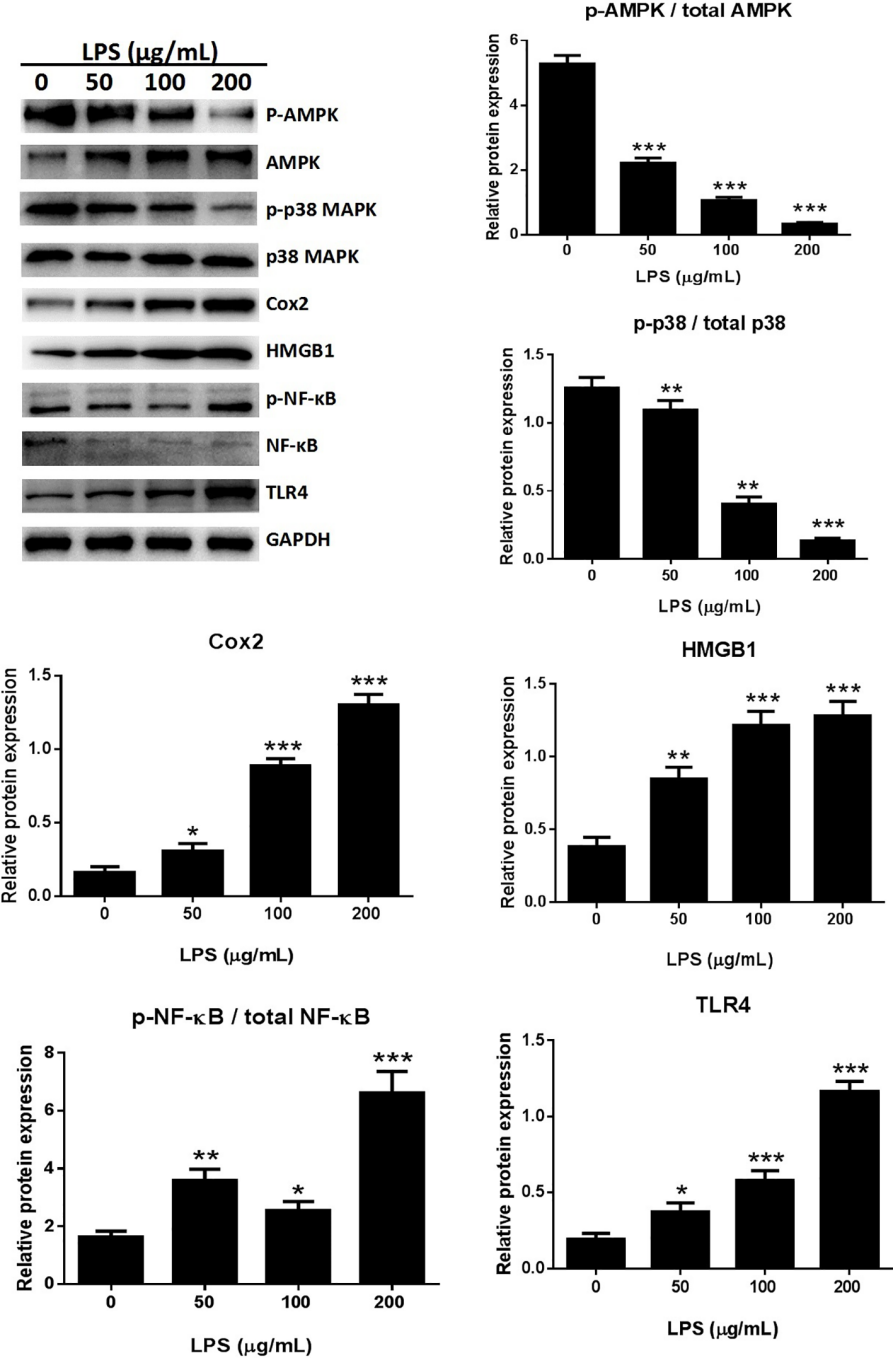
LPS induced the release of inflammatory cytokines through the MAPK/NF-κB/TLR4 pathway. Western blot analysis and quantification of MAPK/NF-κB/TLR4 signaling-related proteins in A549 cells stimulated with various concentrations of LPS for 24 h. *p < 0.05, **p < 0.01, and ***p < 0.001 compared with the control group. Graphs show the mean ± SD of triplicate wells and represent three independent experiments.

The expression of several pro-inflammatory cytokines, including HMGB1 and Cox-2, was significantly increased following treatment with 50, 100, or 200 μg/ml LPS when compared with the untreated control group in an *in vitro* study (**Figure 4**). The possible involvement of TLR4 in the LPS-induced inflammatory process (Li et al., 2018) was assessed by western blotting. LPS treatment dose-dependently increased TLR4 expression in A549 cells (**Figure 4**), suggesting that LPS can activate TLR4 in A549 cells.

NF-κB participates in the inflammatory process as a key transcriptional factor, and the NF-κB pathway is directly affected by activation of TLR4 (Chunzhi et al., 2016; Fan et al., 2016). The effects of LPS treatment on TLR4-mediated NF-κB signaling of inflammatory responses were assessed by western blotting to detect NF-κB and p65 expression. The expression of p-p65 was significantly higher in the LPS treated cells than in the control groups (**Figure 4**). Immunofluorescence assays for detection of nuclear translocation of p-p65 revealed that exposure to 100 μg/ml LPS for 3 h inhibited p-p65 translocation from the cytosol to the nucleus (**Figure 8B**). A previous study found that LPS inhibited NF-κB transcriptional activity by downregulating nuclear p65 levels (Li et al., 2017). Our results also showed that LPS dramatically increased NF-κB activation and dephosphorylation of p65, AMPK, and p38 (**Figure 4**). LPS promoted the release of pro-inflammatory cytokines through the TLR4/NF-κB/MAPK pathway activation.

### rCC16 Improved the Cell Viability Reduced by LPS Treatment

**Figure 5:**
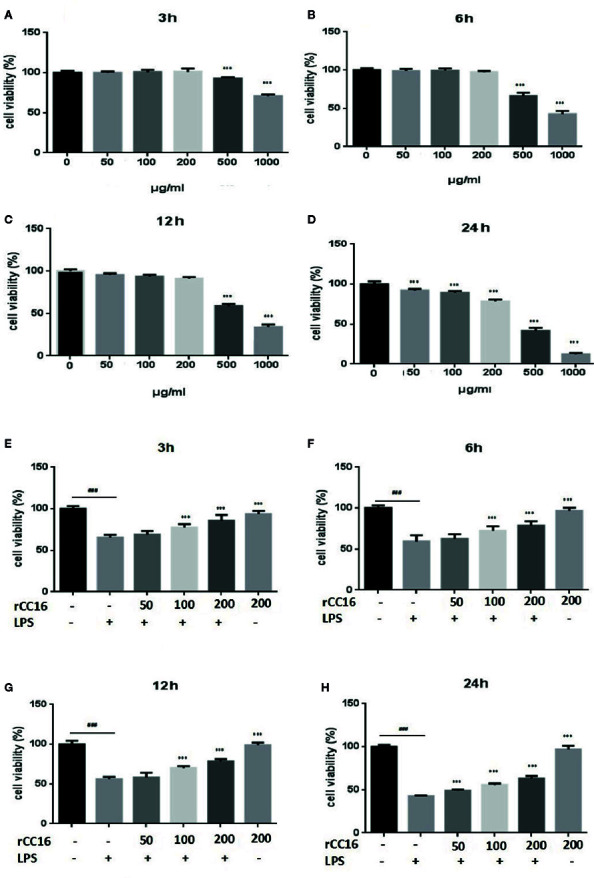
rCC16 attenuated LPS-induced cell injury in A549 cells. **(A)** The effect of rCC16 on the viability of A549 cells for 3 h. **(B)** The effect of rCC16 on the viability of A549 cells for 6 h. **(C)** The effect of rCC16 on the viability of A549 cells for 12 h. **(D)** The effect of rCC16 on the viability of A549 cells for 24 h. **(E)** The effect of rCC16 on LPS-induced cell viability loss for 3 h; cells were pretreated with different concentrations of rCC16 for 12 h and then incubated with or without 200 μg/ml LPS for an additional 3 h. **(F)** The effect of rCC16 on LPS-induced cell viability loss for 6 h; cells were pretreated with different concentrations of rCC16 for 12 h and then incubated with or without 200 μg/ml LPS for an additional 6 h. **(G)** The effect of rCC16 on LPS-induced cell viability loss for 12 h; cells were pretreated with different concentrations of rCC16 for 12 h and then incubated with or without 200 μg/ml LPS for an additional 12 h. **(H)** The effect of rCC16 on LPS-induced cell viability loss for 12 h; cells were pretreated with different concentrations of rCC16 for 24 h and then incubated with or without 200 μg/ml LPS for an additional 24 h. The data are expressed as the mean ± SD; ^###^p < 0.001 compared with the untreated group; *p < 0.05, **p < 0.01, and ***p < 0.001 compared with the LPS group. Graphs show the mean ± SD of triplicate wells and represent three independent experiments.

At concentrations from 0–200 μg/ml, rCC16 protein showed no toxicity to A549 cells after treatment for 3, 6, or 12 h when compared to the untreated control group (**Figures 5A–C**). By contrast, A549 cells treated with rCC16 protein at concentrations of 50, 100, and 200 μg/ml for 24 h showed significantly decreased cell viability when with the untreated control group (**Figure 5D**). As shown in **Figures 5E–H**, the cell viability after exposure to 100 μg/ml LPS resulted in a moderate decrease to about 50%. Therefore this concentration was selected for subsequent experiments. Pretreatment with rCC16 at different concentrations (50, 100, 200 μg/ml) for 3 h alleviated LPS-induced cell damage, as shown in **Figures 5E–H**. After 24 h of LPS treatment, the cell viability was less than 50%, whereas the cell protective effect of rCC16 pretreatment was still significant (p < 0.001) (**Figure 5H**). These results suggested that the LPS-induced decrease in cell viability was inhibited by rCC16.

**Figure 6:**
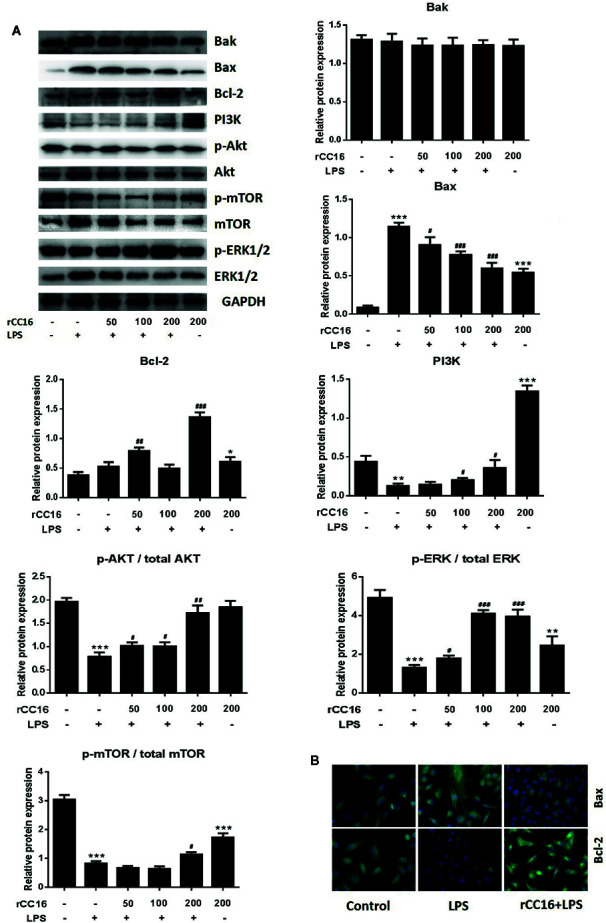
rCC16 reversed LPS-induced A549 cell apoptosis through the PI3K/AKT/mTOR/ERK1/2 pathway. **(A)** Western blot analysis and quantification of apoptosis-related proteins and PI3K/AKT/mTOR/ERK1/2 signaling-related proteins in A549 cells stimulated with various concentrations of LPS for 24 h. **(B)** Immunofluorescence detection of Bax and Bcl-2 after pretreatment with or without 0.2 μg/ml rCC16 for 12 h and treatment with 200 μg/ml LPS for an additional 24 h. ^#^p < 0.05, ^##^p < 0.01, and ^###^p < 0.001 compared with the untreated group; *p < 0.05, **p < 0.01, and ***p < 0.001 compared with the LPS group. Graphs show the mean ± SD of triplicate wells and represent three independent experiments.

The underlying mechanism by which rCC16 inhibits LPS-induced apoptosis was explored by examining the expression of Bcl-2, Bax, and Bak. As shown in **Figures 6A, B**, rCC16 treatment increased the expression of Bax and Bcl-2 but had no significant effect on Bak expression. Therefore, rCC16 appeared to inhibit LPS-induced cell apoptosis by the intrinsic pathway.

### Treatment With rCC16 Reversed LPS-Induced A549 Cell Apoptosis Through the PI3K/AKT/mTOR/ERK1/2 Pathway

The inhibitory mechanism of rCC16 on LPS-induced cell apoptosis was examined by western blotting to examine the protein expression of PI3K, Akt, p-Akt, mTOR, p-mTOR, ERK1/2, and p-ERK1/2. The levels of PI3K, p-Akt/Akt, and p-ERK1/2/ERK1/2 were significantly and dose-dependently enhanced following treatment of LPS-stimulated A549 cells with 50, 100, and 200 μg/ml (**Figure 6A**). The levels of PI3K, p-Akt/Akt, and p-mTOR/mTOR were significantly increased in LPS-stimulated A549 cells, but only by rCC15 concentrations of 200 μg/ml (**Figure 6A**). In summary, pretreatment with rCC16 promoted A549 cell proliferation and inhibited LPS-induced A549 cell apoptosis by activating the PI3K/AKT/mTOR/ERK1/2 pathway.

### Treatment With rCC16 Reversed the LPS-Induced Release of a Variety of Inflammatory Cytokines

**Figure 7:**
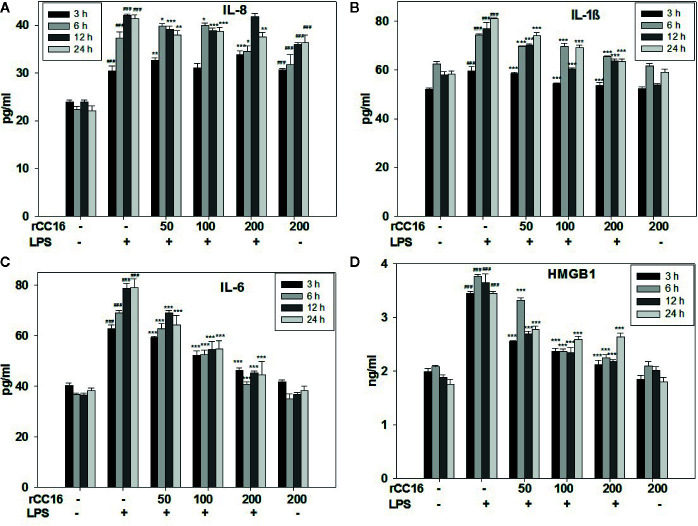
rCC16 reversed the LPS-induced release of the inflammatory cytokines IL-8 **(A)**, IL-1β **(B)**, IL-6 **(C)**, and HMGB1 **(D)** from A549 cells. A549 cells were pretreated with different concentrations of rCC16 for 12 h and then incubated with or without 200 μg/ml LPS for an additional 3, 6, 12, or 24 h. ^###^p < 0.001 compared with the untreated group; *p < 0.05, **p < 0.01, and ***p < 0.001 compared with the LPS group. Graphs show the mean ± SD of triplicate wells and represent three independent experiments.

**Figure 8:**
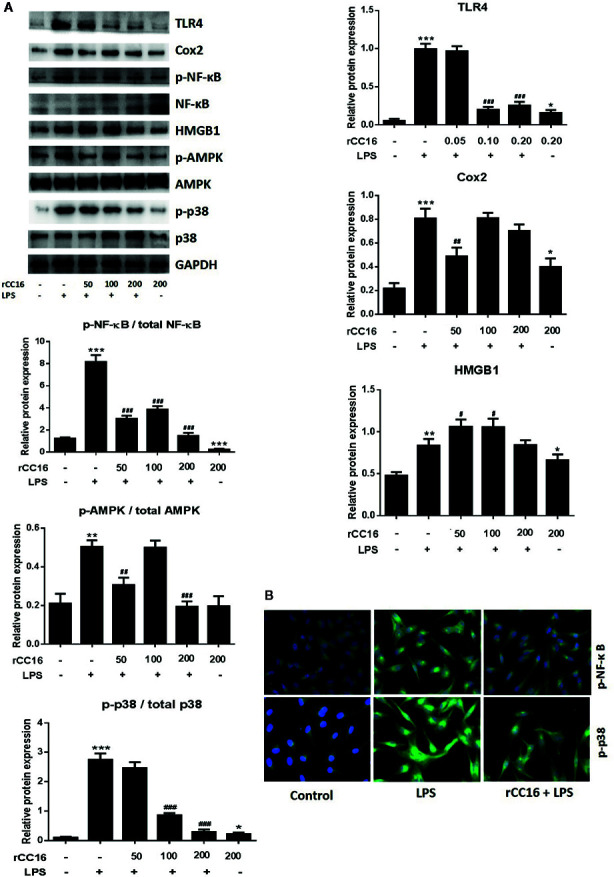
rCC16 suppressed the LPS-induced activation of the TLR4/NF-κB/MAPK pathway. **(A)** Western blot analysis and quantification of apoptosis-related proteins and TLR4/NF-κB/MAPK signaling-related proteins in A549 cells after pretreatment with 0-200 μg/ml rCC16 for 12 h and treatment with or 200 μg/ml LPS for an additional 24 h. **(B)** Immunofluorescence detection of p-NF-κB and p-p38 after pretreatment with or without 0.2 μg/ml rCC16 for 12 h and treatment with 200 μg/ml LPS for an additional 24 h. ^#^p < 0.1, ^#^p < 0.01, ^###^p < 0.001 compared with the untreated group; *p < 0.05, **p < 0.01, and ***p < 0.001 compared with the LPS group. Graphs show the mean ± SD of triplicate wells and represent three independent experiments.

The expression of inflammatory cytokines in A549 cells in response to rCC16 pretreatment was examined by detecting the expression of following inflammatory indicators: HMGB1, IL-1β, IL-6, IL-8, iNOS, COX-2, TNF-α, and SP-C. As shown in **Figures 7A–C**, the expression of IL-1β, IL-8, and IL-6 was increased by treatment with 0.1 mg/ml LPS for more than 3 h, but these increases were significantly reduced by pretreatment with 50, 100, and 200 μg/ml rCC16 (p < 0.001). Cells pretreated with 50, 100, and 200 μg/ml rCC16 showed dose-dependent downregulation of TNF-α expression levels after 24 h of LPS treatment (**Figure S1**). The HMGB1 expression level in cells pretreated with 50, 100, and 200 μg/ml rCC16 decreased significantly compared with a control group at different time points (**Figure 7B**). We also found that different concentrations of rCC16 had no pronounced inhibitory effect on the expression of SP-C, Cox-2, or iNOS (**Figure S1**). Our western blotting results also verified that the protein levels of Cox-2 and HMGB1 were significantly decreased in rCC16-pretreated A549 cells following LPS treatment (**Figure 8A**).

### Treatment With rCC16 Suppressed the LPS-Induced Activation of the TLR4/NF-κB/MAPK Pathway

Western blotting was performed to determine whether rCC16 suppressed LPS-induced inflammation by decreasing the TLR4 expression. TLR4 expression was decreased in A549 cells treated with different concentrations of rCC16 (**Figure 8A**). The expression of NF-κB and p65 was also measured by western blotting to confirm whether rCC16 suppressed the inflammatory responses driven by the TLR4-mediated NF-κB signaling pathway. A significant decrease was observed in the p-NF-κB and p-p65 levels in the rCC16-treated groups compared with the LPS-induced groups (**Figure 8A**). The nuclear translocation of NF-κB was also detected by immunofluorescence assay. Translocation of p-p65 from the cytosol to the nucleus was promoted by exposure to rCC16 (200 μg/ml) for 3 h (**Figure 8B**). Treatment with rCC16 also dramatically decreased NF-κB levels and phosphorylation of and p65, AMPK, and p38 (**Figure 8**). Taken together, these results showed a clearly that rCC16 suppresses LPS-induced inflammatory responses by inactivation of the TLR4/NF-κB/MAPK pathway.

## Discussion

Currently, ARDS therapies are primarily aimed at improving lung-protective ventilation, but are not sufficiently effective, so ARDS treatment remains a continuing challenge in this field (Matthay et al., 2017). Studies of many clinical specimens and animal models have reported that decreases in the CC16 protein content in the airway are an important factor in the occurrence and development of airway inflammation (Guerra et al., 2015). This indicates that CC16 protects lung endothelial cells from damage by inflammatory cytokines, but the molecular mechanisms of CC16 protection against ARDS are still unclear.

ARDS is an inflammatory disorder, and inflammatory cascades play an important role in its occurrence and development. One factor that can induce ARDS is LPS, which acts as a primary infectious stimulus that can lead to severe inflammatory diseases like ARDS (Kovacs-Kasa et al., 2016). The lung injury mediated by LPS shares many characteristics of sepsis-induced ARDS (Marshall et al., 2009), and excessive inflammatory cell infiltration is a characteristic of ARDS (Bhargava and Wendt, 2012). As shown in **Figures 1** and **2**, LPS-treatment of A549 cells significantly reduced cell viability, increased Bax and Bak expression, and decreased Bcl-2 expression. The effects appeared to involve the PI3K/AKT/mTOR/ERK1/2 pathway, which is critical for cell growth and survival, and inhibition of this signaling pathway may lead to cell apoptosis (Ersahin et al., 2015). The levels of p-AKT, p-mTOR, and p-ERK1/2 were significantly decreased in A549 cells after treatment with LPS (**Figure 2**), suggesting that LPS may block mTOR activation through an AKT-dependent pathway. We confirmed that the pathway associated with cell survival was altered in A549 cells after LPS treatment.

The pathogenesis of ARDS and other pulmonary inflammation-related conditions are closely associated with the increased expression of some proteins and cytokines. One example is HMGB1, which is one of the most important chromatin proteins and is secreted by immune cells through the leaderless secretory pathway (Tjalsma et al., 2000). The occurrence of an inflammatory stimulation promotes the secretion of HMGB1 by activated macrophages and monocytes and the HMGB1 then serves as a cytokine mediator of inflammation (Wang et al., 1999). Recent studies have shown that HMGB1 is expressed in a variety of cells in the lungs and its expression is closely related to lung-associated diseases. HMGB1 activates inflammation and plays a role as an alarm in pulmonary inflammation (Gangemi et al., 2015). HMGB1 is an important cytokine that plays a key role in initiating and maintaining inflammatory responses (Huan et al., 2015). One of the most important actions of HMGB1 is to bind to TLR4 to mediate HMGB1-dependent activation of cytokine release (Huan et al., 2010). Studies have shown that the HMGB1-LPS complex that activates TLR4 leads to activation of various signaling cascades (Gangemi et al., 2015). The downstream signaling effect is the activation of MAPK and NF-κB, leading to the inflammatory cytokine production (Hreggvidsdottir et al., 2012).

A correlation also exists between inflammatory responses and regulated cell death (Linkermann et al., 2014). In particular, p38 MAPK plays a significant role in lung inflammation and cell apoptosis and it is activated by LPS (Youssef et al., 2019). Our results demonstrated that the inflammatory cytokines HMGB1, IL-1β, IL-6, IL-8, and TNF-α were increasingly secreted by A549 cells following induction by LPS (**Figure 3**). We sought the relevant mechanism by investigating the NF-κB and p38 MAPK signaling pathways related to inflammation. The protein expression of TLR4, p-NF/κB, MAPK, Cox-2, and HMGB1 was significantly higher in cells treated with LPS than in untreated control group, while the protein expression of p-AMPK, NF-κB, and p-p38 was lower in LPS treated groups than in untreated control group (**Figure 4**). These results suggested that the HMGB1/TLR4/NF/κB and AMPK/p38 MAPK intracellular pathways were activated through phosphorylation.

Clinically, the decreased expression of other proteins and cytokines has also been demonstrated in ARDS and other pulmonary inflammatory diseases. For example, low levels of CC16 in the serum and lung have been linked to the occurrence and development of ARDS (Laucho-Contreras et al., 2018). The purpose of the present study was to investigate the hypothesis that rCC16 can suppress inflammatory responses by reducing HMGB1 expression through the NF-κB and p38 MAPK pathways. This study emphasized the anti-inflammatory and cellular protective mechanism of rCC16 in the PI3K/AKT/mTOR/ERK1/2 pathway and our results confirm that rCC16 can inhibit LPS-induced apoptosis and promote A549 cell proliferation by activating this signaling pathway (**Figure 6**). We verified the protective benefits of rCC16 against inflammation by evaluation the levels of several related inflammatory factors. The ELISA results showed that rCC16 can inhibit the release of certain inflammatory factors, especially HMGB1 (**Figure 7**).

We explored the underlying mechanism of rCC16 inhibitory activity in the LPS-induced expression of HMGB1 and other inflammatory factors by analyzing the protein expressions related to the HMGB1/TLR4/NF/κB and AMPK/p38 MAPK signaling pathways that are activated by LPS. Treatment with rCC16 dose-dependently reversed the phosphorylation and activation of these pathways induced by LPS (**Figure 8A**). Our immunofluorescence staining assays further validated the inhibition of NF-κB and p38 MAPK phosphorylation in A549 cells by rCC16 and the inhibition of cell apoptosis and inflammatory responses (**Figure 8B**). These findings suggest that the protective activity of rCC16 is associated with inhibition of the expression of inflammatory cytokines that are modulated by the HMGB1/TLR4/NF-κB and AMPK/p38 MAPK signaling pathways.

Taken together, the results presented here highlight the potential of rCC16 for the prevention or treatment of inflammation and show that rCC16 may play an essential role in future clinical treatment of ARDS. In clinical practice, increasing evidence now supports the idea that CC16, which has anti-inflammatory and antitoxic properties in the lung, may prevent inflammatory pulmonary disease (Lock-Johansson et al., 2014). The levels of CC16 in the blood and airways also closely track the prevalence and severity of ARDS (Ware et al., 2013). However, thus far, these results have only been confirmed in cells. Whether rCC16 can be used for ARDS prevention and treatment still requires clinical determination, but our study provides a theoretical basis for this possibility.

## Conclusions

In conclusion, our data indicate that LPS might block mTOR activation through an AKT-dependent pathway that induces A549 cell apoptosis and activates inflammatory responses by activating TLR4/NF-κB/AMPK signaling pathways. Pretreatment with rCC16 was confirmed to promote A549 cell proliferation by activating the PI3K/AKT/mTOR/ERK1/2 pathway and inhibiting the release of certain inflammatory factors, especially HMGB1, through dephosphorylation and inactivation of TLR4/NF-κB/AMPK signaling pathways. These results highlight the potential utility of rCC16 for the prevention or treatment of inflammation and show that rCC16 may play an essential role in future clinical treatment of ARDS.

## Data Availability Statement

The raw data supporting the conclusions of this article will be made available by the authors, without undue reservation, to any qualified researcher.

## Author Contributions

We declare that this research work was done by the authors named in this article. Conceptualization: JinL and XZ. Methodology: JieL and MS. Software: WW. Validation: JieL and MS. Formal analysis: JinL., WZ, and QD. Investigation: JieL and JinL. Resources: JW and XZ. Data curation: XX. Writing original draft preparation: JinL and JieL. Writing review and editing: MS and XZ. Visualization: XX. Supervision: WZ and JW. Project administration: WZ and XZ. Funding acquisition: JinL, MS, and XZ. All authors contributed to the article and approved the submitted version.

## Funding

This research was funded by grants from the Natural Science Foundation of China (81503221), the Science and Technology Planning Project of Shenzhen Municipality (JCYJ20180305123707368, JCYJ20180302144713444), and the Natural Science Foundation of Guangdong Province (2017A030313659).

## Conflict of Interest

The authors declare that the research was conducted in the absence of any commercial or financial relationships that could be construed as a potential conflict of interest.
