# Supplementary material for: The rCC16 Protein Protects Against LPS-Induced Cell Apoptosis and Inflammatory Responses in Human Lung Pneumocytes
Source: Front Pharmacol. 2020 Jul 14;11:1060. doi: 10.3389/fphar.2020.01060 (PMC7371929; doi:10.3389/fphar.2020.01060)
Supplement: Supplementary file 1 [file Image_1.pdf]

Supplementary Material

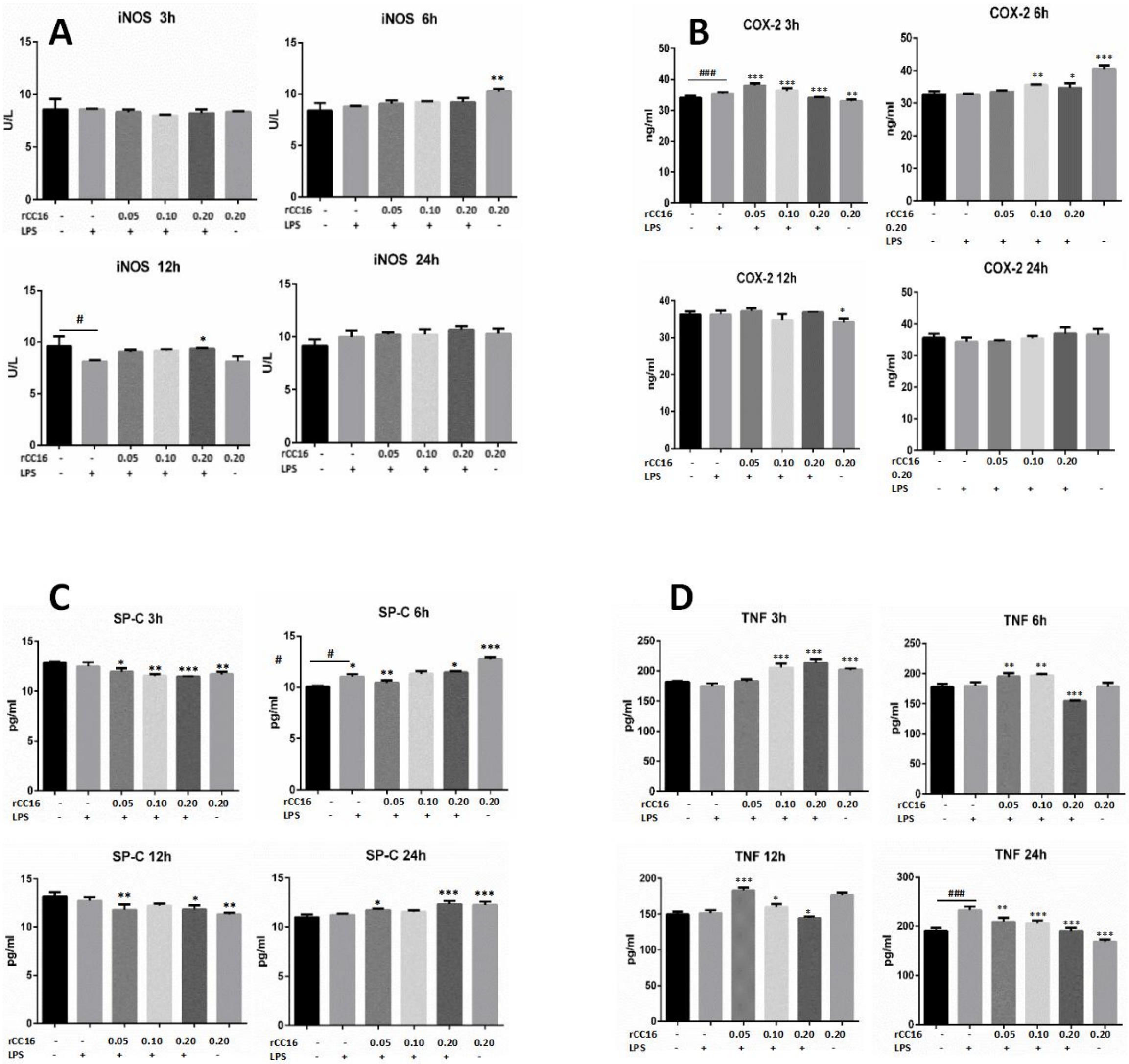

Figure *S*1. Effects of rCC16 on the LPS-stimulated expression of inflammatory cytokines in A549 cells. A549 cells were incubated with rCC16 (0–0.2 μg/mL for 3 h followed by LPS exposure at 200 μg/mL for an additional 24 h. #p < 0.05 and ###p < 0.001 compared with the control group; *p < 0.05, **p < 0.01, and ***p < 0.001 compared with the model group. Graphs show the mean ± SD of triplicate wells and represent three independent experiments.
